# Supplementary material for: The Outcome of Discontinuing Tyrosine Kinase Inhibitors in Advanced Sarcoma Following a Favorable Tumor Response to Antiangiogenics Therapy
Source: J Clin Med. 2022 Dec 31;12(1):325. doi: 10.3390/jcm12010325 (PMC9821264; doi:10.3390/jcm12010325)
Supplement: Supplementary file 1 [file jcm-12-00325-s001.zip › jcm-2086205-supplementary.pdf]

Supplementary Material

Supplementary Figures

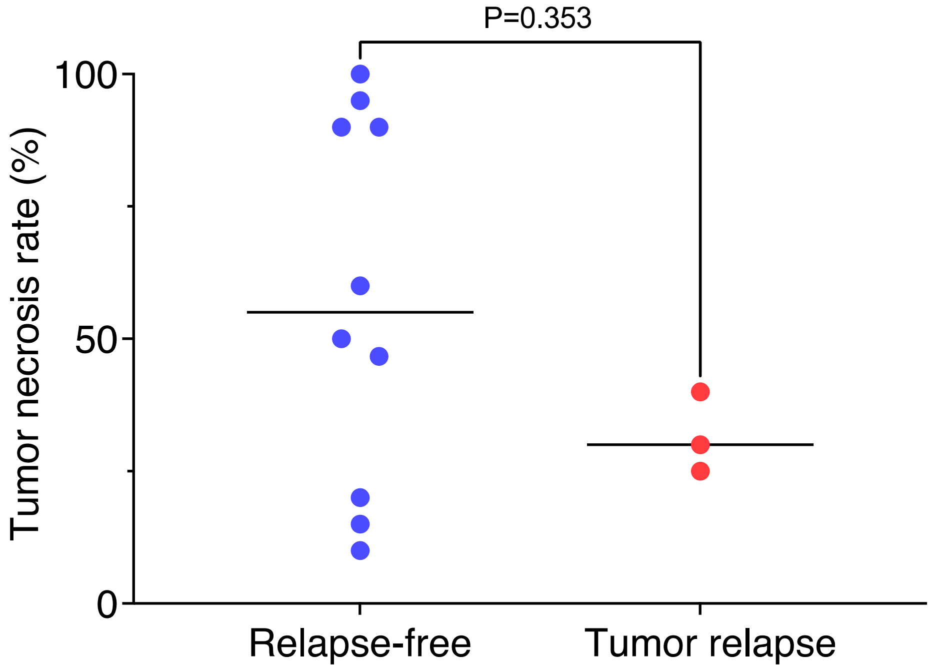

Supplementary Figure S1. The association of tumor necrosis rate with relapse-free survival

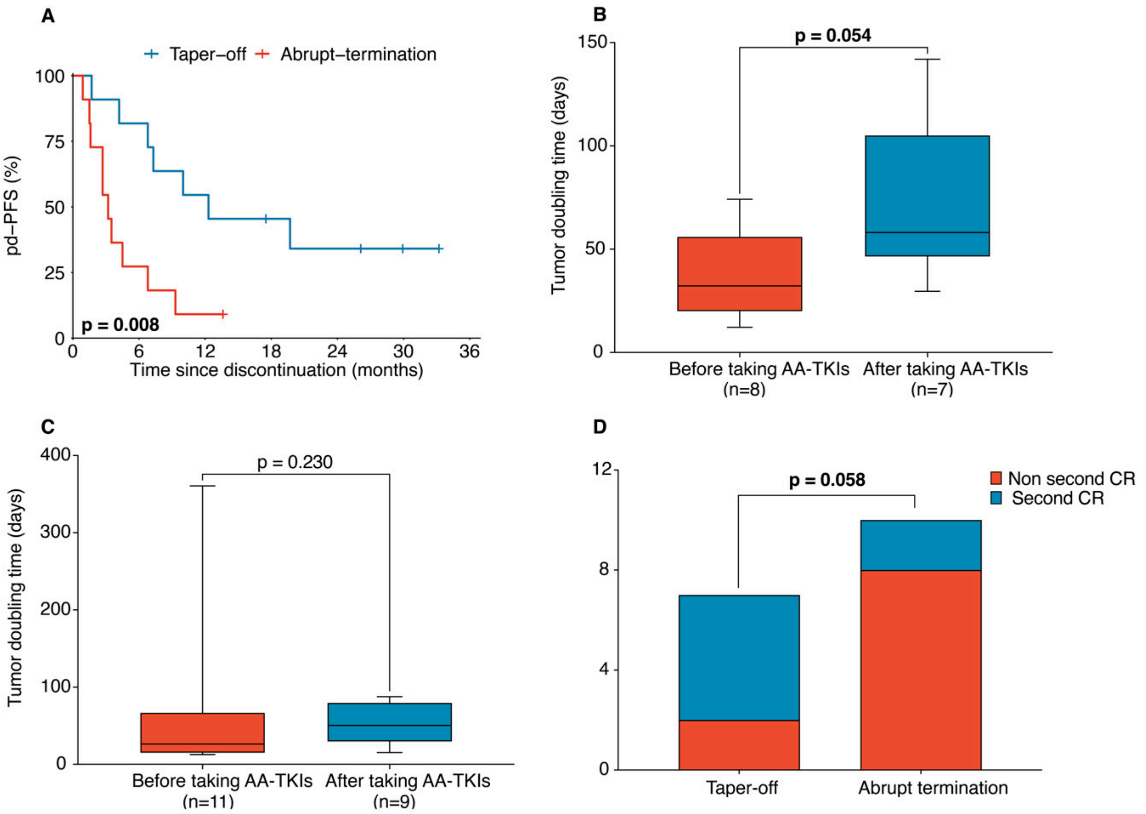

Supplementary Figure S2. The potential benefits of taper-off strategy

Pd-PFS = Progression-free survival since the discontinuation of TKI, TDT = tumor doubling time, AA-TKIs = antiangiogenics tyrosine kinase inhibitors.

(A): Kaplan-Meier curve for pd-PFS. Analysis estimates show the distribution of pd-PFS by TKI discontinuation analysis (n=22). Crosses indicate censored patients. Discontinuation by tapering-off was independently associated with improved pd-PFS (P=0.008).

(B): Comparison of TDT in the taper-off group. Mann-Whitney test showed the distribution of TDT in the taper-off group. (n=8 vs 7). Discontinuation by tapering off was associated with improved tumor doubling time (P=0.054, borderline significance).

(C): Comparison of TDT in abrupt termination group. Mann-Whitney test showed the distribution of TDT in abrupt termination group (n=11 vs 9). Abrupt withdrawal could not prolong tumor doubling time (P=0.230)

(D): Comparison of second CR rate between taper-off and abrupt termination group. Compared to the abrupt withdrawal strategy, taper-off may bring the percentage of second CR (71.4% versus 20.0%, borderline significance, p=0.058)
